# Statin-induced diabetes: incidence, mechanisms, and implications

**DOI:** 10.12688/f1000research.8629.1

**Published:** 2016-06-24

**Authors:** Om P. Ganda

**Affiliations:** 1 Clinical Research and Adult Diabetes Sections, Joslin Diabetes Center, Department of Medicine, Harvard Medical School, Boston, MA, USA

**Keywords:** diabetes, statin therapy, diabetogenesis

## Abstract

Persuasive data from many randomized controlled trials and large, long-term observational studies indicate a modestly increased risk for the emergence of new diabetes after statin initiation. Several meta-analyses of many statin trials as well as longitudinal population-based studies suggest that the risk factors for diabetes in statin-treated persons include underlying risk for diabetes at baseline (specifically features of metabolic syndrome), the intensity of statin therapy, certain genetic traits independent of diabetes risk, and adherence to lifestyle factors. Limited data suggest statins modestly worsen hyperglycemia and A1c levels in those with pre-existing diabetes or glucose intolerance. The precise mechanism(s) of diabetogenesis with statin therapy are unclear, but impaired insulin sensitivity and compromised β cell function via enhanced intracellular cholesterol uptake due to inhibition of intracellular cholesterol synthesis by statins, as well as other mechanisms, may be involved. Furthermore, while statins are known to have anti-inflammatory effects, it is hypothesized that, under dysmetabolic conditions, they might have pro-inflammatory effects via induction of certain inflammasomes. This concept requires further elucidation in the human. Finally, it is clear that the risk–benefit ratio for cardiovascular disease events is strongly in favor of statin therapy in those at risk, despite the emergence of new diabetes. Adherence to lifestyle regimen is critical in the prevention of new diabetes on statins.

## Introduction

The remarkable value of HMG-CoA reductase inhibitors (statins) in atherosclerotic cardiovascular disease (CVD) risk reduction is clearly established, based on landmark secondary intervention as well as primary prevention trials during the past two decades ^1, 2^. The practical significance of a modest absolute risk reduction in primary prevention of CVD is still debated ^3, 4^. However, the recent long-term, global, multi-ethnic, primary prevention trial Heart Outcomes and Prevention Evaluation (HOPE)-3 has confirmed the benefits of a moderate dose of statin (rosuvastatin 10 mg) in subjects at an intermediate CVD risk, with a 24% reduction in primary CVD outcomes (hazard ratio [HR] 0.76; 95% confidence interval [CI] 0.66–0.88; p<0.001) over a mean follow-up of 5.6 years ^5^. These results were consistent with those reported in the meta-analysis of 27 primary prevention statin trials ^2^. While the total mortality or the CVD mortality in the 12,705 subjects in HOPE-3 was not reduced, a significant 15% reduction in CVD mortality and a significant 9% reduction in overall mortality per 1 mmol/L reduction in low-density lipoprotein cholesterol (LDL-C) over ~5 years were reported in the previous meta-analysis of the primary prevention trials ^2^. Based on the overall evidence from the randomized controlled trials (RCTs), various updated major guidelines ^6– 8^ have paved the way for greater attention to initiation and intensification of statin therapy in high-risk individuals (such as those with prior CVD) and in individuals without CVD (such as those with diabetes and multiple risk factors).

## Emergence of new diabetes in RCTs

A clinically relevant concern with statin therapy is a significantly increased risk of new-onset diabetes in patients on statin therapy. The JUPITER trial reported a 25% increase with rosuvastatin 20 mg, over a median follow-up of 1.9 years, compared to those on placebo ^9^. Since then, several meta-analyses have confirmed a smaller but significant increase with various statins ( Table 1). The analysis by Sattar *et al.* in 91,140 subjects showed a 9% overall risk in 13 RCTs over a mean period of 4.0 years (odds ratio [OR] 1.09; 95% CI 1.02–1.17) ^10^. In a subsequent meta-analysis of five intensive-dose statin trials, Preiss *et al.* reported a significant increase in diabetes incidence with more intensive- vs. moderate-dose statin (OR 1.12; 95% CI 1.04–1.22) in 32,752 subjects over a mean follow-up of 4.9 years ^11^. In general, there was no relationship between % LDL-C reduction and incident diabetes. Further analysis of baseline characteristics of the various trials reported a strong relationship between features of metabolic syndrome or pre-diabetes (age, body mass index [BMI], hypertension, fasting glucose, and triglycerides) at baseline and subsequent development of diabetes ^12– 14^.

Of note, the risk–benefit ratio for CVD still clearly favored statin therapy in various studies, including JUPITER, in primary prevention ^13^, several secondary prevention studies ^12, 14^, and a meta-analysis of secondary prevention studies by Preiss *et al.* ^11^. Thus, regardless of whether or not diabetes was diagnosed during statin therapy, the CVD outcomes were reduced on statin therapy compared to those observed with placebo.

**Table 1.** Meta-analyses of randomized controlled trials.

Authors	n	Age (years)	Duration of follow-up (years)	Adjusted odds ratio (95% confidence interval)	Comments
Sattar *et al.* ^10^ 13 trials, statin vs. placebo	91,140	Means: 55.0–76.0	Mean: 4.0	1.09 (1.02–1.17)	Highest risk in older patients; unrelated to % low-density lipoprotein cholesterol reduction
Preiss *et al.* ^11^ 5 trials, more- vs. less- intensive statin	32,752	Means: 58.0–64.0	Mean: 4.9	1.12 (1.04–1.22)	Odds ratio for incident cardiovascular disease 0.84 (95% confidence interval 0.75–0.94)
Navarese *et al.* ^15^ 17 trials, various statins and doses	113,394	Means: 55.0–65.0	2.0–6.0	Pravastatin 40 mg vs. placebo: 1.07 (0.89–1.30) Atorvastatin 80 mg vs. placebo: 1.15 (0.90–1.50) Rosuvastatin 20 mg vs. placebo: 1.25 (0.82–1.90)	Odds ratio unrelated to % low-density lipoprotein cholesterol reduction

Another meta-analysis by Navarese *et al.* is the largest so far: it includes 17 RCTs (more than 113,000 patients). It compared new-onset diabetes in patients receiving statin vs. placebo, or high-dose vs. moderate-dose statins ^15^. The lowest risk was seen with pravastatin 40 mg compared to placebo (OR 1.07; 95% CI 0.83–1.30), whereas rosuvastatin 20 mg was associated with the highest risk (OR 1.25; 95% CI 0.82–1.90) and atorvastatin 80 mg was intermediate (OR 1.15; 95% CI 0.9–1.50), even though none of these differences achieved statistical significance. Simvastatin also appears to be associated with higher risk compared to pravastatin. These differences among various statins persisted after adjustments for reduction in cholesterol. These findings suggest possible molecule-specific effects on diabetogenesis, although the data thus far are inconclusive.

The effects of the newest statin, pitavastatin, are not available in a large enough cohort. In a recent meta-analysis of 15 short-term RCTs of pitavastatin, most of 12 weeks’ duration, total follow-up 1600 person-years, there was no significant difference in the risk for diabetes (OR 0.70; 95% CI 0.30–1.61) compared to placebo ^16^. If confirmed in a larger RCT, it will raise the possibility of differences in pharmacodynamics and drug-drug interactions on diabetogenecity.

## Emergence of new diabetes in population-based, observational studies

Table 2 summarizes several large observational studies ^17– 21^ comparing patients on statins with those not on statins in various populations. These analyses revealed considerable variability among studies and with various statins, with HRs ranging from 1.19–1.57 but statistically significant, after follow-up durations of 3–6 years. In the Women’s Health study, the women were older than several other populations and generally on moderate-dose therapy, yet the HR was 1.48 ^17^. In the largest study of over 2 million subjects in the UK, there was a significant time-dependent increase in diabetes risk (HR 1.57; 95% CI 1.55–1.60), which increased further (HR 3.63; 95% CI 2.44–5.38) in those who were followed for up to 15–20 years ^21^. In one study in patients following myocardial infarction, there was no difference in intensive- vs. moderate-dose statin therapy ^22^, although the CVD outcomes were reduced with the more intensive approach. One caveat with all of the observational studies is that, despite multifactorial adjustments, some differences in the cohort characteristics may not be fully accounted for. In particular, it should be noted that the risk for diabetes according to presence of pre-existing diabetes risk factors, as observed in the several analyses of RCTs ^12– 14^, was not adequately examined in the various observational studies, a major limitation in those studies, compared to RCTs.

**Table 2.** Population-based studies.

Authors	n	Age (years)	Duration of follow-up (years)	Adjusted hazard ratio (95% confidence interval)	Comments
Culver *et al.* ^17^ Women’s Health Initiative (WHI)	153,840	Mean: 63.2 Range: 50-79	3.0	1.48 (1.38–1.59)	Only 7.4% were on atorvastatin, none on rosuvastatin Hazard ratio identical with or without cardiovascular disease at baseline
Cederberg *et al*. ^18^ METSIM (Finnish men)	8749	Range: 43–73	5.9	1.46 (1.11–1.74)	Risk dose dependent for atorvastatin and simvastatin
Castro *et al.* ^19^ Olmsted county, MN	18,071	Range: 43–73	6.0	Normoglycemic: 1.19 (1.05–1.35) Impaired fasting glucose: 1.24 (1.11–1.38)	Mortality reduced in both groups on statin
Corrao *et al*. ^20^ Lombardy, Italy	115,709	Mean: 62.0	6.4	1.12 to 1.32 per statin adherence	
Ko *et al*. ^22^ Ontario, Canada	17,080	Mean: 78.0 Range: 65-78	5.0	Incidence rates for intensive vs. moderate statin: 13.6 vs. 13.0% (non-significant)	Mortality and acute coronary syndrome rates lower with intensive statin: 44.8 vs. 46.5% (p=0.044)
Macedo *et al.* ^21^ UK practice database	2,016,094	Range: 30–85	5.4	1.57 (1.55–1.60)	Hazard ratio increased to 3.63 (95% confidence interval 2.44–5.38) by 15–20 years
Carter *et al.* ^25^ Ontario, Canada	471,250	Median: 73.0	0.5–1.0	Dose dependent compared to pravastatin	Atorvastatin, rosuvastatin, simvastatin > pravastatin

## Glucose control with statins in pre-existing diabetes or abnormal glucose tolerance

There are some observations of interest from a few studies in patients with pre-existing glucose intolerance or diabetes. In the study by Castro *et al.* ^19^, the HR for progression to diabetes was similar in those with normoglycemia, or impaired fasting glucose at baseline, but both groups showed similar reduction in mortality after a 6-year follow-up. In a meta-analysis of nine RCTs in 9696 patients with type 2 diabetes, with a mean follow-up of 3.6 years, there was a modest but significant increase in mean A1c level of 0.12% (95% CI 0.04–0.20) ^23^. In one cross-sectional study in patients with type 1 diabetes (n=1093), statin use was associated with a similar 0.2% increase in mean A1c after multivariate adjustments ^24^.

## Mechanisms underlying diabetogenic effects of statins

The precise mechanism(s) for statin-induced diabetes remain unclear, although the majority of patients developing diabetes have pre-diabetes or features of metabolic syndrome indicating high risk for diabetes at baseline ^12, 13^. It has been controversial whether chemical differences and pharmacodynamic differences in statins or more intensive statin therapy are more likely to precipitate diabetes. In the analysis by Preiss *et al.*, intensive statin therapy led to a greater increase in diabetes ^11^. This was also confirmed in other meta-analyses by Carter *et al.* ^25^ and Dormuth *et al.* ^26^. However, this was not confirmed in a propensity score-matched cohort of patients with myocardial infarction who were prescribed intensive- or moderate-dose statins and followed for 5 years (new diabetes in 13.6 vs. 13.0%) ^22^. The reported lack of new diabetes in pitavastatin-treated subjects is intriguing in view of the relatively small and short-term studies with this newest statin so far (as discussed above) ^16^. Another intriguing observation is that in the fairly large cohort of the HOPE-3 trial (n=12,705), there was no increase in the risk for new diabetes (HR 1.02; 95% CI 0.85–1.23) compared to a 25% increase with rosuvastatin 20 mg in JUPITER ^9^. Whether this relates to the differences in the intensity of statin therapy, risk factors for diabetes at baseline, or perhaps genetic differences in the multi-ethnic HOPE-3 cohort is worth exploring.

Several mechanisms have been postulated underlying the derangements in glucose metabolism by statins. There is some evidence for the detrimental effects of statins on both insulin sensitivity and β cell secretion. In the large METSIM observational study of more than 8000 men, simvastatin and atorvastatin were related to a dose-dependent increase in post-glucose load, an increase in glycemia, a mean decrease in insulin sensitivity by 24%, and a decline in insulin secretion by 12% ^18^. Similarly, in a small study in 28 patients with polycystic ovary syndrome (PCOS), treatment with atorvastatin 20 mg compared to placebo over 6 months led to a decrease in insulin sensitivity despite a decrease in the inflammatory marker C-reactive protein (CRP) ^27^.

A number of potential deleterious effects of statins on β cell function have been proposed, including the effects of increased influx of cholesterol due to inhibition of HMG-CoA-mediated intracellular cholesterol synthesis, inhibition of ubiquinone (CoQ 10) synthesis leading to mitochondrial oxidative stress, and β cell apoptosis ^28^. Statins are generally thought to have anti-inflammatory effects ^1, 9^. However, a recent novel hypothesis posits that under certain conditions, statins may activate inflammasome NLRP3 from macrophages or adipocytes in the presence of endotoxins, leading to interleukin-1β-mediated insulin resistance ^29^. This hypothesis requires confirmation in human studies, as adipose tissue is not a major glucose-metabolizing tissue. A provocative possibility is that the altered gut microbiome, in the presence of obesity or other dysmetabolic states, might provide the endotoxin lipopolysaccharide (LPS) that may mediate the paradoxical pro-inflammatory effect of statins by activation of inflammasomes ^30^. However, under physiological circumstances, a moderate decrease in insulin sensitivity should be compensated for by enhanced insulin secretion by β cells. Thus, ultimately, the direct or indirect effects of statins on β cell function may play an important role in diabetogenesis, particularly in those already at increased risk.

An exciting new observation is that a genetic polymorphism leading to a reduced activity of HMG-CoA reductase is associated with lower LDL-C, a significant increase in body weight, and features of insulin resistance ^31^. This observation was validated in the randomized statin trials, and one particular allele was associated with a significant increase in the risk of new diabetes (OR 1.12; 95% CI 1.06–1.18). Since statins inhibit HMG-CoA reductase as their mode of action, this may at least partly explain their diabetogenic effect.

Finally, a mundane and more simplistic nutritional explanation has been reported by the long-term data in the NHANES study. In a cross-sectional follow-up of more than 27,000 adults followed over 10 years, it was shown that those on statins liberalized their fat and total caloric intake and gained weight over time compared to those not on statins ^32^. Thus, the progression to diabetes could be explained by lifestyle-induced worsening in insulin sensitivity. It is possible that this and the other postulated mechanisms could co-exist.

## Implications of statin-induced diabetes and worsening of hyperglycemia

As summarized above, there appears to be a significant relationship between statin use and the development of new diabetes over the course of several years. This has resulted in an understandable concern about the need to be more vigilant in the use of statins in primary prevention, particularly in those at low absolute risk of CVD. However, the outcome data, particularly from the RCTs, remind us that the subsequent events are also significantly reduced in those with statin-induced diabetes ^9– 14^. Sattar *et al.* calculated, based on their meta-analysis of 13 RCTs, that treatment with statins compared to placebo in 255 subjects over 4 years will cause one new case of diabetes while preventing 5.4 major CVD events ^10^. Similarly, Preiss *et al.* estimated that intensive statin therapy, compared to moderate-dose statins, in those with prior CVD will prevent around three new events per year in ~500 subjects while resulting in one additional case of diabetes ^11^. Finally, Ridker *et al.* reported that in the JUPITER trial, despite a 25% increase in the relative risk for new diabetes with rosuvastatin 20 mg, the major CVD event rate reduction in those who developed diabetes (HR 0.63; 95% CI 0.25–1.60) was consistent with event reductions in the trial as a whole (HR 0.56; 95% CI 0.46–0.69) ^13^. Moreover, the risk of developing diabetes in that trial was almost entirely confined to those with pre-existing features of metabolic syndrome or pre-diabetes ^13^, with similar data from other trials ^12^. Also, in JUPITER, in those with risk factors for diabetes, 134 vascular events or deaths were avoided for every 54 new cases of new diabetes. Finally, in recent data from a cohort of more than 15,000 propensity-matched subjects who initiated statin therapy and were followed for a median of 2.7 years, there was no increase; in fact, there was a significant decrease in the development of microvascular complications (retinopathy and neuropathy) in those developing diabetes compared to a matched non-diabetic cohort ^33^.

Thus, it seems quite clear that statin treatment should not be withheld in those at high risk of CVD for the relatively minor concern of progression to diabetes. In fact, the data described above indicate that in the presence of multiple risk factors for diabetes or metabolic syndrome at baseline in the at-risk population, a modest diabetogenic effect of statin therapy may lead to progression from pre-diabetes to diabetes. This should therefore prompt advice for lifestyle intervention, already known to prevent or delay progression to diabetes, and should be implemented prior to statin initiation. Moreover, the observations from the NHANES survey stated above ^32^, that those on statin therapy generally increased their caloric intake and fat intake leading to progressive weight gain, i.e. factors known to be predictors of diabetes, further emphasize the need for lifestyle counseling as the integral component of both diabetes and CVD event reduction.
